# Motivational interviewing competencies among UK family nurse partnership nurses: a process evaluation component of the building blocks trial

**DOI:** 10.1186/s12912-016-0176-0

**Published:** 2016-09-20

**Authors:** Sue Channon, Marie-Jet Bekkers, Julia Sanders, Rebecca Cannings-John, Laura Robertson, Kristina Bennert, Christopher Butler, Kerenza Hood, Michael Robling

**Affiliations:** 1 South East Wales Trials Unit, Centre for Trials Research, Cardiff University, Neauadd Meirionnydd, Heath Park, Cardiff, CF14 4YS UK; 2 School of Healthcare Sciences, Cardiff University, Cardiff, UK; 3 Department of Psychology, Bath University, Bath, UK; 4 Primary Care Clinical Epidemiology, University of Oxford, Oxford, UK

**Keywords:** Motivational interviewing, Family nurse partnership, NFP, MITI, Building blocks trial, Home visiting

## Abstract

**Background:**

Motivational Interviewing (MI) is a person-centred counselling approach to behaviour change which is increasingly being used in public health settings, either as a stand-alone approach or in combination with other structured programmes of health promotion. One example of this is the Family Nurse Partnership (FNP) a licensed, preventative programme for first time mothers under the age of 20, delivered by specialist family nurses who are additionally trained in MI. The Building Blocks trial was an individually randomised controlled trial comparing effectiveness of Family Nurse Partnership when added to usual care compared to usual care alone within 18 sites in England. The aim of this process evaluation component of the trial is to determine the extent to which Motivational Interviewing skills taught to Family Nurse Partnership nurses were used in their home visits with clients.

**Methods:**

Between July 2010 and November 2011, 92 audio-recordings of nurse-client consultations were collected during the ‘pregnancy’ and ‘infancy’ phases of the FNP programme. They were analysed using The Motivational Interviewing Treatment Integrity (MITI) coding system.

**Results:**

A competent level of overall MI adherent practice according to the MITI criteria for ‘global clinician ratings’ was apparent in over 70 % of the consultations. However, on specific behaviours and the MITI-derived practitioner competency variables, there was a large variation in the percentage of recordings in which “beginner proficiency” levels in MI (as defined by the MITI criteria) was achieved, ranging from 73.9 % for the ‘MI adherent behaviour’ variable in the pregnancy phase to 6.7 % for ‘percentage of questions coded as open’ in the infancy phase.

**Conclusions:**

The results suggest that it is possible to deliver a structured programme in an MI-consistent way. However, some of the behaviours regarded as key to MI practice such as the percentage of questions coded as open can be more difficult to achieve in such a context. This is an important consideration for those involved in designing effective structured interventions with an MI-informed approach and wanting to maintain fidelity to both MI and the structured programme.

**Trial registration:**

Current Controlled Trials ISRCTN23019866 Registered 20/4/2009.

## Background

The importance of increasing health enhancing behaviours such as physical activity and reducing unhealthy behaviours such as smoking and excessive alcohol consumption is well established in reducing the risk of developing chronic disease and overall disease burden in the general population [1]. Individual behaviour change is a key component in making the lifestyle adjustments needed to reduce these risks. Motivational Interviewing (MI), “a person-centred counselling style for addressing the common problem of ambivalence about change” (P29) [2] has been increasingly used by a range of health professionals in their practice across a range of settings, including primary care and public health, to engage their patients and clients in thinking about change.

MI comprises four broad processes; engaging, focusing, evoking and planning, all within the context of a collaborative relationship in which the client’s autonomy is fully accepted by the practitioner. MI is often used as a stand-alone intervention but also as an adjunct to other approaches, in particular to promote engagement with an intervention [2, 3].

There has been one recent systematic review of MI in primary care [4] and two meta-analyses of randomised controlled trials of MI in medical settings (including primary care, hospital and community health) [5, 6]. An increase in health-enhancing behaviours shown either through self-report or objective measures in relation to physical activity, dietary intake or alcohol consumption were noted in approximately 50 % of studies in primary care. [4]. However, this systematic review highlighted the inconsistencies and lack of clarity in the various descriptions of the MI components of the interventions. In a meta-analysis of studies using MI as the primary component of the intervention with primary care populations, the mean effect sizes were described as being largest in outcomes related to weight loss, blood pressure and substance use [5]. In the review of randomised controlled trials (RCTs) in medical care settings the overall effect showed a significant modest advantage of MI with particular strength in areas such as HIV, dental outcomes, weight, alcohol and smoking. The strongest effects were in evidence when MI was compared to waiting list control groups but there was also a significant effect when compared to treatment as usual or information-only interventions. Each provider type produced positive outcomes, although only the results from mental health providers and mixed teams were reported to reach statistical significance [6]. However, determining the nature of the MI content was a common evaluation challenge which therefore affects interpretation of many reviewed studies. MI may be integrated with other intervention components and few studies assess fidelity of MI delivery e.g. only 14 % of the studies included in the meta-analysis [6] reported on the measurement of fidelity.

With MI often included in the communication skills component of training healthcare practitioners and interventions often described as including MI, it is important to establish firstly if practitioners can learn the MI skills but also then integrate MI competencies into their routine practice. As outlined in a systematic review of training health professionals in MI [7], clinicians are often inaccurate in their self-evaluation of MI skills, both over and underestimating their MI ability, so we need to measure this more objectively. It is also clear that whilst MI skills acquisition has been demonstrated in a range of studies [7] there are also examples where it has been found to be difficult to then use MI skills in routine practice [8]. Therefore examination of practice in a range of service contexts and cultures using the same measures is needed.. This study describes one structured, manualised programme which has incorporated MI into the staff’s core training, Family Nurse Partnership, and measures the extent to which the nurses’ intervention delivery demonstrates MI competencies using an established measure of MI fidelity.

### The Family Nurse Partnership

The Family Nurse Partnership in England (FNP) [9] is a licensed, preventative programme for first time mothers under the age of 20, developed in the US as the Nurse-Family Partnership (NFP) [10]. It offers intensive structured home visiting, delivered by specially recruited and trained nurses, from early in pregnancy until the child is aged two, covering three distinct phases of pregnancy, infancy, and toddlerhood.

A scheduled maximum of 64 visits (14 during pregnancy, 28 during infancy and 22 during toddlerhood) cover core content areas of personal and environmental health, life course development, maternal role, family and friends and access to health and social services. The exact number of visits will be determined by individual need and engagement and by gestational age at enrolment. Visits are supported by FNP manuals which provide a structure and recommended content for each visit.

Family nurses use the manual, materials and methods to enable young mothers (and fathers) to achieve three main aims; i) to improve pregnancy outcomes including achieving a healthy birthweight by changes such as reducing smoking, ii) to improve their child’s health and development by developing their parenting knowledge and skills and iii) to improve parents’ economic self- sufficiency, by helping them to achieve their aspirations (such as employment or returning to education).

The programme draws upon three guiding theoretical perspectives; Human Ecology [11] Bandura’s Self-efficacy theory [12] and Bowlby’s Attachment theory [13] with a defined conceptual model representing how the programme elements act together to influence maternal and child health development [14] and a theory of change logic model [15]. Specified within the UK FNP management manual are core model elements (CMEs) and fidelity goals which collectively represent the mechanism used to ensure fidelity to the programme model. CMEs are licensing requirements intended to ensure replication of the original US research conditions. They relate to both programme delivery and infrastructure requirements and specifically address client enrolment and engagement; nurse recruitment, training and working practices; supervisor recruitment, training and working practices; administrative support; implementing agencies. The license to deliver FNP in the UK depends on sites achieving these CMEs and licence fees are paid annually.

In the US three large randomised controlled trials with long-term follow-up provide empirical support for the claims of the success of the programme [16]. Outcomes assessed in the trials include maternal health, rates of child injury, abuse and neglect, and, when the children reach adolescence, anti-social behaviours and mental health problems [17–23].

FNP has been delivered in England since 2007, following adaptation and with initial testing in ten sites [24]. Programme capacity was increased to 16,000 places across more than 130 English sites and it is also available in parts of Scotland and Northern Ireland [25]. FNP teams comprise up to eight nurses, each of whom carries a maximum caseload of 25 clients who they usually work with throughout their contact with FNP, a supervisor who carries a reduced caseload, and an administrator.

### Motivational interviewing and FNP

In the US client retention and completion of home visits was lower in programme delivery than in the original trials of NFP, with considerable variation between sites. Sites with the lowest levels of participant retention were also those where nurses used more directive, prescriptive approaches to working with clients. In contrast, nurses at the low attrition sites more often adapted the programme to clients’ needs [26]. This finding led to the introduction of the principles of Motivational Interviewing (MI) into the intervention to enhance engagement and reduce attrition. In the pilot study of MI in NFP across 17 sites in the US [27] the nurses were trained to address parents’ ambivalence about participation and to offer flexible scheduling and content to match their needs. The results showed that an MI-based modification to the NFP programme showed promise as a way of reducing attrition and increasing completed home visits.

From the outset FNP England has included skills training in MI delivered by members of Motivational Interviewing Network of Trainers (MINT) in the training and on-going supervision of the family nurses (see Table 1 for training details).

**Table 1:** Training in Motivational Interviewing received by FNP teams during the Building Blocks trial

Initial training in MI: A two- day workshop covering the core principles and skills of MI using presentation, role play and skills practice including asking open questions, affirmations, reflective listening, summarising, agenda setting and planning for change.
Supervisor training: Two days training on incorporating MI skills into their supervisory practice and to develop the supervisors’ skills and confidence to lead the post-workshop skills development sessions. Focus on collaboration, evocation, autonomy support, elicit-provide-elicit, using role play of small group practice.
Post workshop: •Four half-day team sessions once every 3 weeks for the nurses to consolidate their learning. Lead by supervisor with materials developed by workshop trainer. Mainly small group practice using real-life scenarios. Sessions content included i) relationship and agenda matching ii) ambivalence and goal setting iii) avoiding righting reflex iv) developing change talk and affirmations. •Skills development day with the trainer to consolidate this learning.

### The Building Blocks trial

Building Blocks is an individually randomised controlled trial within 18 sites in England comparing the effectiveness of FNP plus usually available care to usual care alone (Trial registration number ISRCTN23019866) [28]. Primary endpoints were tobacco use by the mother at late pregnancy (using self-report calibrated with an objective measure of cotinine in urine samples to determine exposure to tobacco smoke), birthweight of the baby, the proportion of women with a second pregnancy within 24 months post-partum, and emergency attendances and hospital admissions for the child within 24 months post-partum. Between June 16, 2009, and July 28, 2010, 3251 women were screened and 1645 women were recruited, with five subsequently excluded due to non-eligibility (one woman was deemed not to be Gillick competent, one woman was identified as not pregnant at the first scan, and three women were registered with a GP outside the study area) The total number of valid visits for all women recruited to receive FNP was 27,853 provided by 106 family nurses and 25 supervisors. The median number (range) of visits clients received in pregnancy phase across all sites was 10 (0–20), in infancy phase was 19 (0–44) and 13 (0–37) in toddlerhood phase.

The Building Blocks trial is the first UK randomised controlled trial of FNP and was reported in 2015 [29]. It included an integrated process evaluation work stream, developed to enable a detailed understanding of the content and process of the programme as it is delivered and to contextualise the findings of the trial. This report describes an analysis of consultations between family nurses and their clients to determine the extent to which Motivational Interviewing skills taught to the family nurses were used in their home visits with clients.

## Methods

The aim of this study was to explore the extent to which the MI skills taught to FNP nurses were deployed in audio recorded consultations. The study was approved by the Wales NHS Research Ethics Committee (09/MRE09/08) and received governance approval from all participating NHS sites. Informed consent was given by the nurse and client for each recording and anonymity for both was retained through the use of ID numbers for each recording.

### Participants

We aimed to have recordings from randomly selected nurses and, based on the size of the teams and their caseloads, we asked two randomly selected family nurses in each of the 18 trial sites to submit a minimum of two recorded consultations each per phase; pregnancy, infancy and toddlerhood. The nurses were eligible for selection for this recording task if they had been delivering the programme for at least 6 months, had received the FNP training in MI and had clients in the appropriate phase at each wave of data collection.

### Data collection

Data were collected by the nurses onto encrypted digital audio recording devices. The nurses were free to determine which clients to approach and were told they could record as many consultations as they wished and select what they considered were the two most representative examples in each phase.

Most consultations in the FNP are conducted in the client’s home and other people such as partners, family members, friends may be with the mother and child during the visit (if this occurred during the recorded sessions they were also asked to consent to the recording). The visit is likely to be over an hour (the average length of visit across the three phases ranged from 73 to 79 min). The whole consultation was recorded and additional analysis (not reported here) was undertaken to review the content of the consultation, with reference to the content domains specified within the FNP programme.

### The motivational interviewing treatment integrity scale

The recordings were analysed using the Motivational Interviewing Treatment Integrity Scale (MITI) [30, 31], to measure integrity to the method of MI through the coding of practitioner utterances.

The MITI (version 3.1.1) has two components (see Table 2).

**Table 2:**
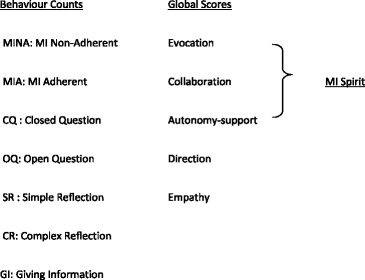
Individual behaviour counts and global scores on the MITI

i) Behaviour counts, which tally seven specific behaviours as defined by the rating scale’s manual without judgement of quality or intent: MI Non-adherent (MINA) (e.g. advising without permission or confronting the client), MI adherent (MIA) (eg affirming the client or emphasising their autonomy). In contrast to simple reflections, complex reflections add substantial meaning, conveying a deeper or more complex aspect of what the client has said, for example if the client said “I am just so angry I can’t believe what they did” the practitioner might say “you’re furious about this” (simple reflection) or “this behaviour is unacceptable to you” (complex reflection).
ii) Global scores based on the rater’s overall impression of how well the practitioner’s approach fits with the intent of each of the five dimensions (see Table 3) during the consultation segment being coded. Each dimension is rated 1–5, specific anchors for the scores are given in the manual (see Table 3). In addition, there is also a score for “MI spirit” a summation of three of the five global scores.

**Table 3:** Global scale anchors for achieving top score of 5 on each scale (reproduced with permission from MITI 3.1.1 manual)

Global Scale	Anchors for scoring maximum of 5 on the scales
Evocation	Clinician works proactively to evoke client’s own reasons for change and ideas about how change should happen
Collaboration	Clinician actively fosters and encourages power sharing in the interaction in such a way that client’s ideas substantially influence the nature of the session.
Autonomy support	Clinician adds significantly to the feeling and meaning of client’s expression of autonomy, in such a way as to *markedly expand client’s experience of own control and choice*
Direction	Clinician exerts influence on the session and generally does not miss opportunities to direct client toward the target behavior or referral question.
Empathy	Clinician shows evidence of deep understanding of client’s point of view, not just for what has been explicitly stated but what the client means but has not yet said.

There have been attempts to understand the mechanisms of change to guide the search for the practitioner behaviours key to client change. The work on identifying specific behaviours has yielded equivocal results [32] and is ongoing in the MI research field. The most consistent finding in a review summarising the evidence for these mechanisms was that MI – inconsistent behaviours (which would be coded as MI Non-Adherent – MINAs) were associated with poorer client outcomes [32]. Similarly, a study of the influence of MI counsellor skills concluded that the overall MI approach (use of MI-consistent skills and avoiding use of MI inconsistent practices) of practitioners is most important [33].

Therefore, in reviewing the findings of the MITI coding, the key foci in terms of fidelity and impact was on the global clinician rating (capturing the “gestalt”), MI adherence and non-adherence: The other individual behaviour counts were collected to provide information on the skills implementation of the nurses in the context of delivering the FNP programme.

### Validity and reliability

The MITI coding is conducted on 20 min segments of the recorded interaction, and the start-time of the segment was selected by using a random number generator. The coding team comprised five coders from a range of backgrounds; two qualitative researchers (HP, MJB), one research assistant (CL), one administrator (KA) and one clinical psychologist (SC), who attended a two-day workshop in the MITI coding system run by two MINT trainers, (NG, JC). The coders then developed their coding skills through individual practice on sample recordings and attendance at regular coding meetings Inter-rater reliability was estimated using the intra-class correlation coefficient (ICC) alongside 95 % confidence intervals (CIs) for the overall scores [34]. The single-measure ICC was used to assess rater agreement to find out whether we can assume that the judgment of one rater is the same as that of the others. Coding skills practice was continued until “excellent” levels of inter-rater reliability, i.e. above 0.75 [35] were achieved on all the variables which took four post-workshop group coding sessions. In addition to the starting measurement, inter-rater reliability on the coding system was measured twice during the coding. Reliability at the end of the coding was also measured against an independent coder, external to the Building Blocks coding team (one of the MITI trainers JC), to ensure fidelity to the coding system. Overall 20 % of the recordings were double-coded.

## Results

### Nurse eligibility and total number of recordings submitted

A total of 61 nurses were eligible to submit recordings across the 18 sites at the planned start of the data collection with the range being 2–6 nurses per site. By the time the pregnancy recording period began, one site no longer had any trial clients in the pregnancy phase so no recordings were collected from this site in either phase. Due to a lack of clients in the designated phase receiving visits from the randomly selected nurses who were willing to be recorded, all eligible nurses were asked to submit recordings if they had eligible and willing clients. Overall 42 (69 %) nurses submitted recordings for either one or both phases.

Pregnancy phase: A total of 48 recordings of home-visits during the pregnancy phase were received from 17 sites. Recordings were submitted by 31 nurses (range of 1–3 nurses per site). The average number of recordings received per site was 3 (range 1 to 5). The average recording length was 73 min (range 46–91 min). On one recording the randomly selected 20-minute segment consisted entirely of a DVD being played so it was excluded from the analysis resulting in 47 to analyse.

Infancy phase: A total of 45 recordings from the infancy phase were received from 16 sites for analysis (the nurses in the other two sites did not have any clients in the infancy phase during the recording period). Recordings were received from 30 nurses and the average number of recordings received per site was 3 (range 1 to 5). The average recording length was 64 min (range 24–98 min).

Toddlerhood phase: We found increasingly complex verbal interactions in the toddler phase recordings, with three-way and nurse-child/mother-child communication that could be ascribed to the developmental stage of the child. The MITI was developed for assessing dyadic interactions so as a consequence of the changes we decided not to analyse the toddlerhood phase recordings.

The recordings for the phases were collected sequentially ie pregnancy then infancy so the data is presented separately for the phases. This would allow for future analysis of the relationship between the domain coverage in the phases and use of MI but also further examination of any impact of time since initial training, practice, developing expertise etc.

### Coder inter-rater reliability

A two-way mixed effects model with single measure ICC was used to assess inter-rater reliability. On the 19 (21 %) recordings that were double coded inter-rater reliability was “excellent” [35] with ICC coefficients ranging from 0.83 to 0.99 for both global scores and behaviour counts across the two phases of recordings coded, with coding of Evocation having the lowest ICC co-efficient and Direction having the highest. At the completion of coding the overall inter-rater reliability with an external independent coder (JC) was 0.83 (95 % CI = 0.62 to 0.97).

### Practitioner behaviours

In order to describe the nurses’ practice in terms of the behaviours used in the MITI the frequency of each behaviour as a proportion of all counted behaviours is summarised in Table 4. The number of specific behaviours (e.g. MI non-adherent) divided by the total number of behaviours for that individual is calculated. What is presented in the table is the median, IQR, range of these proportions across all individuals. The relative frequency of individual behaviours was the same across both phases: Closed Questions were the most common type of utterance (33.3 % pregnancy phase and 30.2 % infancy phase) followed by Giving Information (30.6 % in pregnancy phase and 23 % infancy phase).

**Table 4:** Frequency of behaviour as a proportion of all counted behaviours (ranked from lowest to highest)

Behaviours	Pregnancy Phase (*N* = 47)			Infancy Phase (*n* = 45)		
	Median (%)	IQR (%)	Range (%)	Median (%)	IQR (%)	Range (%)
MI Non-Adherent	0.0	0.0 to 1.0	0.0 to 7.3	0.0	0.00 to 0.5	0.0 to 7.69
MI-Adherent	5.1	2.8 to 7.4	0.0 to 14.8	4.0	1.7 to 6.4	0.0 to 12.0
Complex Reflections	5.8	2.5 to 9.5	0.0 to 16.7	5.6	4.0 to 9.6	0.0 to 18.9
Open Questions	9.8	6.23 to 15.3	3.7 to 26.4	7.9	3.2 to 15.0	0.0 to 33.0
Simple Reflections	16.0	9.3 to 20.4	2.0 to 30.9	22.0	17.8 to 28.6	11.1 to 39.7
Giving Information	30.6	19.1 to 37.3	5.4 to 50.7	23.0	16.8 to 32.8	6.2 to 54.3
Closed Questions	33.3	25.2 to 40.6	8.0 to 59.8	30.2	24.4 to 37.1	13.3 to 49.3

### Practitioner competencies variables

In addition to the five global scores and counts of the seven behaviours, a number of derived practitioner competency variables (Table 5) were created by the MITI authors [31] to capture skilful MI practice (e.g. the balance between questions and reflections which is central to skilful MI). The authors also generated suggested levels for practitioner competencies in MI for these derived variables (Table 5). Levels 2 and 3 are the MITI manual thresholds for ‘beginning proficiency’ and ‘competency’ thresholds respectively whilst level 1 indicated when scores did not reach the first threshold of ‘beginning proficiency’ in MI.

**Table 5:** Practitioner Competency: Derived Variables and proficiency and competency thresholds

Variables created	Components of the variables	“Beginners proficiency” Level 2	“Competency” Level 3
Global Clinician Rating	Average of the 5 global scores	Average of 3.5	Average of 4
Reflections: Questions ratio	Total reflections/total questions	1	2
% Open Questions	OQ/(OQ + CQ)	50 %	70 %
% Complex Reflections	CR/(CR + SR)	40 %	50 %
% MI-Adherent	MIA/(MIA + MINA)	90 %	100 %
Total Questions	CQ + OQ	-	-
Total Reflections	SR + CR	-	-
MI spirit	Average of scores on Evocation, Collaboration and Autonomy support	-	-

As shown in Table 6 the median rate of MI adherence was 100 % in both phases and the global clinical rating (with potential rating range 0–5) was 4.0 in pregnancy and 3.8 in infancy. In the pregnancy phase there were also twice as many total questions as total reflections asked (median ratio 0.5). In the infancy phase the median reflection: question ratio had increased to 0.7. In the pregnancy phase a median of 25.0 % of all questions were coded as Open and a median of 26.7 % of all reflections were coded as Complex. In the infancy phase the percentage of open questions and complex reflections dropped slightly from the pregnancy phase to 18.8 % for both variables. The MI adherence/non-adherence competency variable had a median of 100 % in both phases reflecting a high rate of adherence compared to MI non-adherent behaviour.

**Table 6:** Descriptive statistics of clinical competency

Derived Variable	Pregnancy phase (*N* = 47)			Infancy phase (*N* = 45)		
	Median	IQR	Range	Median	IQR	Range
Global Clinician Rating	4.0	3.6 to 4.2	2.6 to 4.8	3.8	3.4 to 4.1	2.6 to 5.0
Reflections: Questions ratio^a^	0.5	0.3 to 0.8	0.1 to 1.5	0.7	0.5 to 1.2	0.4 to 2.0
% Open Questions	25.0	16.1 to 34.5	5.8 to 66.7	18.8	11.8 to 32.0	0.0 to 66.7
% Complex Reflections	26.7	18.2 to 40.0	0.0 to 66.7	18.8	13.3 to 33.3	0.0 to 46.7
% MI-Adherent^b^	100.0	85.7 to 100.0	0.0 to 100.0	100.0	80.0 to 100.0	16.7 to 100.0
MI spirit	3.7	3.3 to 4.0	2.0 to 5.0	3.3	3.0 to 4.0	1.7 to 5.0

^a^ *a* value <1 indicates a higher number of total questions than total reflections, value >1 indicates a higher number of total reflections than total questions. ^b^ *N* = 46 for % MIA as one home-visit scored MIA = 0 and MINA = 0 and *N* = 38 for % MIA in infancy phase as seven visits scored 0 on both MIA and MINA

Table 7 shows the number and proportion of visits from the self-selected recordings in which the family nurse's practice meets the thresholds for MI proficiency (level 2) or competency (level 3). The scores placed them at level 2 proficiency or higher on the global clinician rating in 38 (80.9 %) of the recordings in the pregnancy phase and 32 (71.1 %) in the infancy phase. On the MI adherent scale there were 34 (73.9 %) recordings with scores at level 2 or above in pregnancy phase and 32 (71.1 %) in the infancy phase. In the pregnancy phase there was no evidence of level 2 proficiency in most of the recordings for the variables based on specific behaviours i.e. the reflection: question ratio, percentage of open questions, and percentage of complex reflections. The infancy phase proficiency scores were similar with the exception of the reflections to questions ratio in which 15 (33.3 %) showed level 2 proficiency, more than double the percentage (14.9 %) that achieved level 2 in the pregnancy phase and in the percentage of complex reflections with 7 (14.9 %) achieving level 3 competency in the pregnancy phase but none achieving level 3 in the infancy phase.

**Table 7:** Number and proportion of visits in which the family nurse meets the level 1, 2 and 3 thresholds for proficiency

	Pregnancy phase (*N* = 47)			Infancy phase (*N* = 45)		
	Level 1	Level 2	Level 3	Level 1	Level 2	Level 3
Global Clinician Ratings	9 (19.1)	13 (27.7)	25 (53.2)	13 (28.9)	14 (31.1)	18 (40.0)
Reflection Questions ratio	40 (85.1)	7 (14.9)	0 (0)	30 (66.7)	15 (33.3)	0 (0)
% Open Questions	42 (89.4)	5 (10.6)	0 (0)	42 (93.3)	3 (6.7)	0 (0)
% Complex Reflections	35 (74.5)	5 (10.6)	7 (14.9)	39 (86.7)	6 (13.3)	0 (0)
% MI-Adherent^a^	12 (26.1)	1 (2.2)	33 (71.7)	11 (28.9)	0 (0)	27 (71.1)

^a^ *N* = 46 in pregnancy phase as one home-visit scored MIA = 0 and MINA = 0 and *N* = 38 in infancy phase as seven visits scored 0 on both MIA and MINA

## Discussion

We aimed to examine the level of competency in MI skills family nurses displayed in the recorded consultations, as measured by the MITI. We found that on the global clinician rating over 70 % of the ratings were at “beginner’s proficiency” level or above. On MI adherence, the ranges are large but the median was 100 % with the majority of recordings evidencing level 3 competency. The current knowledge from the findings of previous research and reviews, suggest that global ratings and MI inconsistent behaviours appear to be most important in assessing impact on outcomes [32]. Based on these measures of fidelity the nurses demonstrated skilfulness in key aspects of MI practice.

The individual behaviour counts provide detailed information on MI implementation within the structure of FNP programme. The two most frequent behaviour codes are related to information exchange i.e. Closed Questions (used to seek specific information) and Giving Information. This compares, for example, to a study of nurse practitioners delivering Motivational Enhancement Therapy to improve diabetes control [36] where the most frequent codes were Complex Reflections and Closed Questions. We expected that Giving Information would be a frequent behaviour, given the high informational component of the FNP programme. The key issue with giving information in an MI consistent manner is the way in which it is done. Specifically, does the client feel they were having advice imposed on them or do they experience it as receiving information they were seeking, with the practitioner having carefully established that they were interested in receiving that information and that choices were given? One measure of this is the levels of “MI non-adherent” codes as this incorporates advice-giving without seeking permission. Given that “MI non-adherent” was the least common behaviour code, information exchange in these consultations was conducted in this more collaborative way in the majority of cases, which is consistent with the MI approach.

The use of reflection and the balance of reflection to questions are central to MI practice. Reflections represented less than a third of coded behaviours in both phases and most were simple rather than complex, which reduces the rating of practitioner competency in this aspect of practice. In terms of the ratio between questions and reflections, only a minority of consultations contained the 1:1 ratio required for beginning proficiency level as defined by the MITI manual. It might be difficult to achieve more reflections than questions, given the high number of questions that are an essential part of the intervention (e.g. health-related questions). This is one of the areas where having a structured intervention may well impact on the nature of the utterances, making it more difficult for the nurses to achieve the ratios recommended for skilful MI practice. Future analyses of the interactions in the recordings with higher ratios could provide examples of how this is achieved. However, increasing the use of reflections would be the clearest first step in enhancing the MI competency of the nurses. This could initially be targeted at simple reflections as a first step with an aim to increase the complexity of the reflections as the nurses gained in confidence and skilfulness.

The reliability of the raters in our study was excellent with a consistent team who were well trained and maintained a good level of scrutiny with ICCs at several points, double coding, use of an external “gold standard” and regular group meetings.

The FNP nurses had received significant training input in MI, combining didactic teaching, coaching and supervision on casework to enable the training to be integrated into practice, which has been shown to be the most effective training model in MI [37]. Two systematic reviews in MI training [7, 37] and a meta-analysis [38] indicate that whilst it is possible to increase MI skilfulness through workshop training, ongoing input is required through more focused client-based consultation and coaching to sustain those skills over time. Without post-workshop support effects of training start to erode by 3 months [39] and efforts to enhance workshop structures, such as more tailored training to fit the context, did not extend skills maintenance. However post-workshop input including coaching, spreading training out and increasing frequency of input to more than an additional 5 h contact time did sustain skills at the six- month follow-up (with few studies looking beyond 6 months).

In applying the MITI (designed for rating MI interventions and coaching MI practitioners) to FNP data, we set the bar high and the levels of competence in MI achieved using the MITI manual definitions were relatively low. However, participating nurses were focused on delivering a structured, complex intervention with a lot of informational content and their primary focus would be on fidelity to the FNP programme. Furthermore, competency levels detailed in the MITI manual are for guidance and based on expert opinion rather than evidence linking MITI rated proficiency to client outcomes beyond the findings on global ratings discussed earlier. In a study in which one social worker worked with people with Multiple Sclerosis to improve exercise experience [40], the mean reflection: question ratio (2.6) and percentage of open questions (56.7) are significantly greater than in our study. Compared to a study in which four graduate practitioners worked with substance misuse [41] there were some similarities (e.g. mean reflection question ratio was 0.72) although the use of complex reflections and open questions was higher than in our study. One of the key differences between the FNP programme and the work in these two studies is the extent to which MI is the intervention focus; in FNP the MI is integrated with another programme so it can be described more as an approach, in the other studies MI is either an additional component of the intervention delivered separately (in the exercise study) or the main intervention approach (in the substance misuse study). In both these cases the interventionist would most likely describe themselves as primarily delivering MI which could have a significant impact on their approach to their delivery. Another key difference is the strong focus on information sharing within FNP which potentially puts the FNP nurse in a more didactic, directing role for a significant part of their contact. This adds to the challenge of using an MI-based approach but is a common situation with many programmes having a substantial information/educative component. Given what is known about the impact of MI non-adherent practice this is a challenge that needs to be addressed in health care communication.

There are difficulties in comparing findings as studies have used different versions of the MITI, there have been concerns raised about cultural differences in language impacting on coding [41] and the majority of studies using the MITI have tended to be in the context of alcohol and substance misuse interventions so the body of evidence in other domains is still relatively small. However, as the number of studies across contexts grows the body of work using the MITI will become substantial enough to start making meaningful comparisons and allow a more in depth examination of both the effectiveness of training on practice and on the relationship between MI practice and client outcomes.

This evaluation will inform endeavours to integrate MI principles into FNP. The high global clinician ratings are reassuring. However, the types of questions asked, the level of reflection and the frequency of complex reflections may be a focus for future training. Our analysis was not intended to assess individual nurse’s competency, the impact on client outcomes, the timing of the recordings in relation to that practitioner’s training, or the phase of delivery. Thus it is not possible to determine whether the slight differences seen between phases are important. However, it may raise an area of development for the teams to be thinking about planned maintenance and development of MI skilfulness over time and how the supervision can address this. Frequency of MI supervision may also be relevant. This may be weekly as reported by Smith et al. [40], and Maissi et al. [36] in a health context or fortnightly as reported in a study of a smoking cessation service [42]. The training in MI in the FNP has changed since this evaluation such that the MI trainers are now part of the national central FNP training team. Further work is needed within FNP to understand the impact of MI training on the practice of FNP nurses and how this might impact on client outcomes within the programme.

### Limitations

The analysis can only reflect the material presented, and a degree of reluctance to expose their practice for evaluation [43]. was anticipated. For example, the average rate of consultation recording return was less than 50 % in two other studies of MI [44, 45] which could raise concerns about the representativeness of the content and quality of the recordings. With a 65 % return rate from 69 % of eligible nurses in this study the submission rate is comparatively high. There were practical considerations such availability of opportunities to record that affected return rates. Whilst nurses were initially randomly selected to submit recordings, difficulties matching nurse and client willingness and availability with the appropriate recording period meant that sites were asked to invite all eligible nurses to submit recordings if they had eligible and willing clients. The FNP nurses were asked to select recordings that they felt best represented their work and they were aware of the purpose of the task so we might assume the recordings represent a picture of ‘best practice’ in MI terms, However, client availability, willingness to consent and nurse anxiety, may also have biased the sample of submitted recordings in ways that are hard to identify e.g. it could be their estimation of ‘best practice’, ‘easiest client’ ‘least amount of communication’. One possibility that might need to be considered in light of the high Giving Information content is that the least complex consultations (in terms of interpersonal content) may have been those that the client consented to have recorded and that the nurses submitted for analysis and those consultations could simultaneously be the most information-driven. There is wide variability in the length of sessions and whilst the procedure for coding a randomly selected 20 min of the session was in accordance with the MITI coding manual, it does mean that there will be sections of sessions that are included for coding that are not optimal for coding MI proficiency such as the more structured parts of the programme delivery. One option that would have improved our understanding of the nurses MI competence would have been to use a role play with a standardised patient and that could then have been compared to their use of MI skills in their FNP routine practice.

The main Building Blocks trial results were published in 2015, showing that adding the Family Nurse Partnership programme to usual care provided no additional short-term benefit for the selected primary outcomes (smoking in pregnancy, birthweight, emergency hospital attendance and admission for the child, and subsequent pregnancy). There is a follow-on study currently in progress which will assess the programmes’ impact on longer-term benefits for mothers and children, looking at outcomes up to 4 years later. One of the main reasons for introducing MI into the intervention was to reduce attrition. The fidelity goals for the programme included attrition rates (≤10 % during pregnancy phase, ≤20 % during infancy phase, ≤10 % during the toddler phase) and all the attrition rates in the trial were well below those levels (3.6 % pregnancy, 10.1 % infancy and 7.9 % toddlerhood). Whilst it is not possible to attribute these high rates of retention to the inclusion of MI in the intervention, they are a strong indicator of the quality of client engagement by the nurses.

## Conclusions

Family Nurses in FNP have demonstrated ability to consult according to principles of MI. Our results indicate that some behaviours and derived variables measured by the MITI may not always be entirely consistent with the structure and context of particular programmes, while the spirit of the approach might well be. This analysis is the starting point with much more to be gleaned about the inter-personal processes of client-nurse communication. It provides feedback that can be incorporated into supervision and skill development. Also with the knowledge of the growing evidence that practitioner behaviours and communication style relate to client outcomes [32, 33], it is important to take a broader perspective on the database of recordings which could enable a detailed examination of the impact of different nurses’ approaches to the blending of information exchange with highly skilled communication. This would be of relevance in many different contexts in responding to health and social care challenges which require the delicate balance of expert knowledge and complexity of agendas, particularly as those interventions are taken to scale.

## Abbreviations

CQ: Closed question
CR: Complex reflection
FNP: Family nurse partnership
GI: Giving information
MI: Motivational interviewing
MIA: MI adherent
MINA: MI non-adherent
MINT: Motivational interviewing network of trainers
MITI: Motivational interviewing treatment integrity
NFP: Nurse-family partnership
OQ: Open question
SR: Simple reflection
